# 6MWT Performance and its Correlations with VO_2_ and Handgrip Strength in Home-Dwelling Mid-Aged and Older Chinese

**DOI:** 10.3390/ijerph14050473

**Published:** 2017-04-29

**Authors:** Qing Zhang, Hailin Lu, Shiqin Pan, Yuan Lin, Kun Zhou, Li Wang

**Affiliations:** School of Nursing, Medical College, Soochow University, Suzhou 215006, China; 20154231012@stu.suda.edu.cn (Q.Z.); jpyoyo03@163.com (H.L.); 20155231008@stu.suda.edu.cn (S.P.); Althea1874@126.com (Y.L.); 20144231008@stu.suda.edu.cn (K.Z.)

**Keywords:** walking distance, muscle strength, graded exercise test, VO_2_, respiratory exchange ratio

## Abstract

Six-minute walk test (6MWT) performance is more commonly used in clinic patients with chronic cardiopulmonary diseases but not in home-dwelling individuals of similar age, and its correlations with oxygen uptake (VO_2_) and muscle strength require further investigation. The current study determined the 6MWT performance of 106 home-dwelling residents (mean age of 62 years) in Suzhou, China. VO_2_ at a respiratory exchange ratio (R) of 1 was measured through graded cycling exercise tests on 46 participants. Handgrip strength of all participants was tested. 6MWT distance measured 543.4 ± 67.2 m (total work 351.0 ± 62.8 kJ) with similar distances ambulated each minute. Heart rate, blood pressure, and rate of perceived exertion scores significantly increased after 6MWT. VO_2_ at R = 1 reached 1238 ± 342 mL/min (18.6 ± 4.7 mL/kg/min), whereas handgrip strength totaled 29.8 ± 9.6 kg. 6MWT distance showed strong correlations with VO_2_ (*r* = 0.549, *p* ≤ 0.001) and handgrip strength (*r* = 0.359, *p* < 0.001). Aside from providing reference values for 6MWT performance (~543 m, ~559 m in males and ~533 in females) for home-dwelling Chinese residents, our results suggest that as a parameter of exercise endurance, 6MWT performance correlates with both aerobic capacity and muscle fitness.

## 1. Introduction

The six-minute walk test (6MWT) is commonly used to measure physical motor function and endurance exercise capacity. As a reliable, inexpensive, safe, and readily available method, 6MWT performance better represents daily physical activities compared with other walk tests [1]. Crucial indicators for 6MWT include measured outcomes of patients before and after treatment of moderate to severe heart and lung diseases [2,3]. However, data from different same-age populations of these patients are essential reference values for clinical and research applications. These values may help us develop realistic expectations regarding improvement of 6MWT in patients undergoing exercise rehabilitation programs.

Several studies investigated 6MWT distance in healthy individuals in Austria [4], the US [5,6,7], and Europe [7,8]. Additional studies investigated 6MWT distance in healthy Asian individuals in Japan [9,10] and Singapore [11]. In China, previous studies on 6MWT have mainly focused on cardiopulmonary patients with a few exceptions of studies on healthy young individuals [12,13]. Thus, insufficient data are available on distances covered by healthy mid-aged and older Chinese individuals after 6MWTs. However, aside from being impractical, difficulty arises from finding healthy mid-aged or older subjects without any health problem or histories of diseases. Therefore, to obtain essential references for clinical patients, data must be collected from the independent and home-living adults without diseases that require hospitalization.

As an evaluation modality for functional exercise capacity, 6MWT distance may correlate with other physical fitness aspects, including cardiopulmonary fitness, which is represented by volume of oxygen uptake (VO_2_). Significant correlations between 6MWT distance and VO_2_ are observed in patients with chronic obstructive pulmonary diseases [14] and in patients with advanced heart failures [15], whereas such results may differ in young healthy individuals [12]. Tests regarding maximal oxygen uptake (VO_2max_) or peak oxygen uptake (VO_2peak_) requires participants to exercise until near exhaustion; thus, such tests pose potential difficulties for older individuals, especially those who seldom exercise or feature chronic diseases. Ventilatory anaerobic threshold (VAT) is a more preferred index of endurance capacity [16] and correlates more with exercise performance [17]. Common indicators of VAT include nonlinear increase in ventilation and carbon dioxide output (VCO_2_) and an increase in the respiratory exchange ratio (R) [17,18]. However, definite breakpoints in these indicators cannot be determined, especially for individuals with poor fitness, and data interpretation can be quite subjective [19,20]. R represents the ratio between VCO_2_ and VO_2_; this ratio is assumed to be the most accurate factor for assessing subjective effort during cardiopulmonary exercise testing [21]. Thereby, the present study uses the criterion that R remains consistently near above 1 as a cut point of graded exercise tests to avoid asking participants to work longer with additional effort to identify the breakpoint of nonlinear change in ventilation (see Section 2.4 in Materials and Methods for the detailed protocol). Average VO_2_ at R = 1 is expected to be similar to VAT and we aim to confirm its correlation with 6MWT distance.

While cardiovascular capacity is a distance-limiting factor, muscle weakness impedes waking; this condition is a common complication in elderly individuals and patients. Although lower limbs are more relevant to walking than upper limbs, in most cases, measurement of their strength becomes harder because it requires special training and equipment (which can be too heavy to move). Handgrip strength is extensively used as an acceptable and simple measurement conducted with a hand dynamometer [22]. This variable also correlates well with leg strength [23]. Both 6MWT distance [24] and handgrip strength [25,26] are strong predictors of mortality. However, further investigations should still unveil the relationship between these two parameters.

The present study aims to examine 6MWT performances of home-dwelling mid-aged and older Chinese individuals to obtain a reference value for clinical patients and to determine the correlation between 6MWT distance and VO_2_ and between 6MWT distance and handgrip strength.

## 2. Materials and Methods

### 2.1. Participants and Ethical Considerations

Participants included 106 (44 males and 62 females) home-dwelling individuals aged between 45 and 90 years old from communities around Soochow University in Suzhou, China; they were randomly recruited by posting flyers on bulletin boards and distributing flyers in communities. The recruitment featured no specific regulation on sex. More females were recruited than males as old women in China more frequently gather in public areas of their communities and participate more in activities than men, who mostly prefer to stay inside their homes and be alone.

Participants were evaluated based on a self-designed questionnaire to ensure that they could walk alone safely and presented no exercise contraindications. The questionnaire was composed of questions on basic information (age, sex, body weight, height, and body mass index (BMI)), health condition (family history, genetic history, injury history, disease history, and current disease information), and exercise habits in the year prior to study (exercise style, duration, frequency, and intensity). Resting heart rate (HR) and blood pressure (BP) were measured and recorded. To represent actual situations, participants included populations that had a history of disease but were currently stable or only manifested minor health problems but were able to exercise without any complications. Exercise habit and history of diseases or health problems of subjects are shown in Supplementary Materials. All participants provided their written consent to express their agreement to participate after being completely informed of purposes and risks of all procedures. This research project was approved by the Human Research and Committee of Soochow University and conducted in compliance with the guidelines stated in the World Medical Association (WMA) Declaration of Helsinki (Project identification code: ECSU-201700025).

### 2.2. Procedure

Participants were instructed to refrain from vigorous exercise 24 h before 6MWT while retaining their usual lifestyle. They were instructed to maintain their usual dietary habits until the day before and during examinations. Caffeine, alcohol, and strong tea were prohibited for at least 2 h before tests. Participants were also instructed to wear comfortable clothes and shoes for walking.

On the test day, HR, BP, and Borg’s 6–20 rate of perceived exertion (RPE) scale of participants were measured and recorded after resting for 10 min. Handgrip strength was tested twice using a hand dynamometer according to manufacturer’s instruction; the higher value from each measurement was recorded. Thereafter, 6MWT distance was measured in all participants. Among all participants, 46 participants finished the graded exercise test to determine their VO_2_. Graded exercise was performed after a 20-min rest on the same day. Several reasons contributed to the considerably fewer participants joining in the VO_2_ test. First, VO_2_ testing required subjects to spend more time and travel longer distances to access the test equipment in the laboratory. Second, most subjects were not interested because of unfamiliarity. Some subjects worried about their incompetence in cycling and were afraid of embarrassment. Despite the complete explanation provided, only 46 out of 106 participants volunteered to undergo the VO_2_ test.

### 2.3. 6MWT

The 6MWT procedure was performed in accordance with guidelines published by the American Thoracic Society [3]. Owing to unavailability of an indoor walkway, tests were carried out on a 30 m outdoor walkway, whose length was marked every 3 m with brightly colored cones. All tests were performed during days with favorable weather. The person who conducted the test used standard Chinese language to instruct and encourage participants at specified times after each minute. All participants received the same instructions. Approximately 15 s before test conclusion, participants were reminded to concentrate on finishing it. Before and immediately after the test, HR, BP, and RPE score of each subject were measured and recorded. None of the participants used walking aids, such as crutches or canes. Details of the procedure can be found in the work of Monte et al. in their Supplementary Material Section (http://jcn.sagepub.com/supplemental) [27].

### 2.4. Graded Cycling Exercise Test

The graded cycling exercise test was performed using Quark PFT system (COSMED, Rome, Italy) with Ergoselect 100 (Ergoline GmbH, Germany). After system calibration, the participants were instructed to sit on the cycle ergometer. Each subject wore an exercise face mask connected to a flow meter to measure breath-by-breath pulmonary gas-exchange variables including VO_2_ and VCO_2_. Selection of a suitable sized face mask for each subject was prioritized and carefully checked to ensure that each mask fitted the wearer without air leaks. After a 3-min warm-up with low-power exercise (30 W for males, 20 W for females), cycling work rate was increased by 10 W in each subsequent minute. Participants were instructed to maintain the pedal speed at approximately 60 rpm. Exercise intensity was increased until the R of each subject was consistently higher than 1. Intensity was then decreased to low power (same as in warm-up) and continued for 2 min as a cool down. Approximately 4–7 min (depending on fitness level of each subject) passed before R reached and remained consistently above 1 from the starting workload after the 3-min warm-up. The procedure for the graded exercise test was adopted based on previous studies [18,28] and on trial tests in our lab on mid-aged or older people with or without chronic diseases (unpublished data). Throughout the entire process, HR was monitored continuously, whereas BP was monitored intermittently. VO_2_ data of five continuous time points at R = 1 were averaged.

### 2.5. Handgrip Strength Test

After adjusting the handle of the hand dynamometer to 0 kg, each participant held the device with their dominant hand, with arms at their sides, and the scale plate facing out. The base was carefully positioned on the first metacarpal (heel of palm), whereas the handle was positioned on middle of four fingers. Participants were strongly encouraged to squeeze the dynamometer with their maximum isometric effort and to keep squeezing for 5 s. No other body movement was allowed. The handgrip strength test was performed twice on each subject, and the higher value was recorded and analyzed.

### 2.6. Statistical Analysis

Each result, unless otherwise indicated, was presented as average (AVE) ± standard deviation (SD). Data were analyzed using SPSS 16.0 software package (IBM, Armonk, NY, USA). Paired-samples *t*-test was applied to examine differences between tested and predicted distances based on previously published equations, and HR, BP, and RPE values before and after 6MWT. Independent-sample t-test was used to compare differences between male and female subjects. One-way ANOVA was used to test differences between distances ambulated at each minute. Correlations between different parameters were analyzed with bivariate correlation analysis. A 6MWT distance prediction equation was obtained from stepwise multivariate linear regression analysis.

## 3. Results

### 3.1. Participants

Table 1 presents the general information for the 106 subjects. All results in this paper were obtained from them except for VO_2_, which was tested on only 46 participants (as explained above).

### 3.2. 6MWT

All participants finished the 6MWT without any unexpected premature termination. The 6MWT distance measured 543.4 ± 67.2 m (375–756 m) with a total work equal to 351.0 ± 62.8 kJ and both parameters were larger in males (558.6 ± 74.1 m, 379.2 ± 63.7 kJ) than in females (532.5 ± 60.2 m, 331.0 ± 54.3 kJ, *p* = 0.048 and *p* < 0.001 respectively). Distances walked for each minute (~89–92 m) were similar. Based on the classification by Bittner et al. [29], 12 (~11%) participants walked between 375–450 m (level 3), and the remaining 94 (~89%) subjects walked more than 450 m (level 4). HR, systolic BP (SBP), diastolic BP (DBP), and RPE scores significantly increased at post 6MWT (*p* < 0.01) (Table 2).

After calculation using the equations previously published by Poh et al. [11], Enright and Sherrill [5], Troosters et al. [8], and Gibbons et al. [6], predicted distances were 452.6 ± 61.2 m, 493.8 ± 65.1 m, 583.2 ± 62.6 m, and 639.5 ± 42.0 m, respectively. Our tested 6MWT distances were larger than those predicted using Poh et al.’s [11] and Enright and Sherrill's equation [5], but smaller than those based on Troosters et al.’s [8] and Gibbons et al.’s equations [6] (*p* < 0.001).

Using stepwise multivariate linear regression analysis of our own data, the obtained 6MWT distance prediction equation is as follows: 6MWT distance (m) = 3.162 × handgrip strength (kg) − 2.538 × age (years) − 1.978 × weight (kg) + 737.4; the equation includes age, handgrip strength, and body weight. The predicting model explained 39.6% of total variance in 6MWT distances (Table 3).

### 3.3. VO_2_

A total of 46 subjects (21 males and 25 females) participated in the VO_2_ test. At R = 1, VO_2_ of 46 participants reached 1238 ± 342 mL/min, with 1417 ± 387 mL/min in males and 1087 ± 208 mL/min in females. Relative VO_2_ measured 18.6 ± 4.7 mL/kg/min (20.3 ± 5.2 mL/kg/min in males and 17.1 ± 3.6 mL/kg/min in females). Both VO_2_ (*p* = 0.001) and relative VO_2_ (*p* = 0.016) were significantly larger in males than in females. HR at R = 1 equaled 121.2 ± 15.3 beats/min, which was equivalent to 74.7% ± 8.5% of the maximum HR (HR_max_) calculated by age.

### 3.4. Handgrip Strength

Handgrip strength reached 29.8 ± 9.6 kg in all participants. This variable totaled 37.7 ± 8.4 kg in males and 23.8 ± 5.1 kg in females, demonstrating a significant difference between sexes (*p* < 0.001).

### 3.5. Correlations of 6MWT Distance with VO_2_ or Handgrip Strength

**Figure 1:**
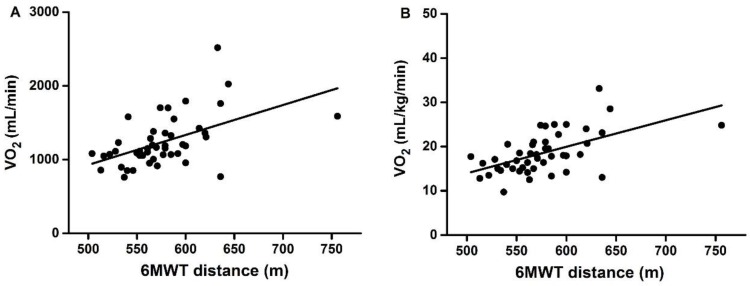
Correlation between 6MWT distance and VO_2_ (**A**) or relative VO_2_ (**B**) at R = 1. R: respiratory exchange ratio.

**Figure 2:**
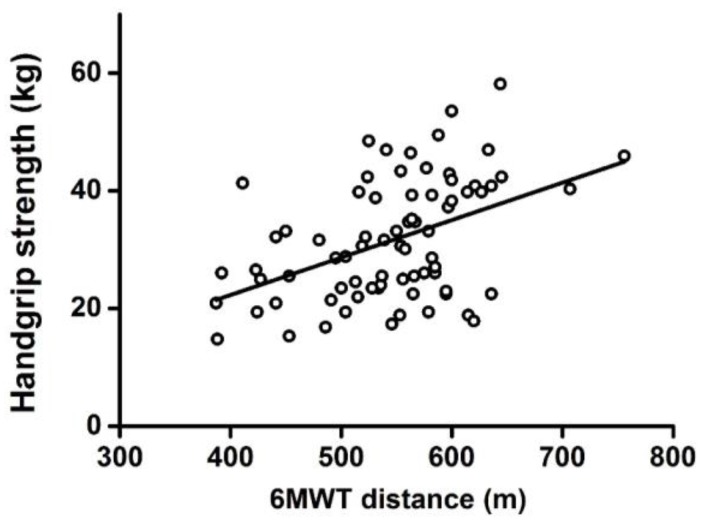
Correlation between 6MWT distance and handgrip strength.

A significant positive correlation was observed between 6MWT distance and VO_2_ (*r* = 0.549, *p* < 0.001) (Figure 1A) or between distance and relative VO_2_ (*r* = 0.591, *p* < 0.001) (Figure 1B). 6MWT distance was also positively correlated with handgrip strength (*r* = 0.359, *p* < 0.001) (Figure 2).

After multivariate partial correlation analysis, results showed that, with sex, age, height, and body weight as control variables, significant positive correlations existed between 6MWT distance and handgrip strength (*r* = 0.221, *p* = 0.029, *n* = 106), between 6MWT distance and VO_2_ (*r* = 0.413, *p* = 0.010, *n* = 46), and between 6MWT distance and relative VO_2_ (*r* = 0.440, *p* = 0.006, *n* = 46).

## 4. Discussion

In the present study, the 6MWT distance deduced from 106 home-dwelling mid-aged and older Chinese volunteers reached 543 m and correlated with handgrip strength. During graded cycling exercises, at R = 1, tested VO_2_ among 46 subjects also correlated with 6MWT distance.

In the present study, 6MWT distance measured 543 m. This level is similar to findings observed in Singaporean adults of similar age (61.0 ± 8.3 years, with initial and secondary test results of 524 ± 95 and 560 ± 105 m) [11] and in independently living European older men (69.1 ± 5.0 years, ~538 m to ~591 m before exercise intervention) [30]. However, this level is larger than results observed in independently living older South American populations (60–79 years, ~438.6 m to ~481.3 m) [31] but lower than those observed in a study by Camarri et al. [4] on healthy individuals in Australia (55–75 years, 659 ± 62 m). Several equations were developed in previous studies to calculate 6MWT distance of healthy subjects [5,6,8,11], and predicted values for our subjects based on these equations were higher or lower, depending on the equation used, than our measured 6MWT distance.

By comparing our study with findings referred to above, we observed that apart from age, body size, and sex of subjects, the testing method also possibly influenced differences in results, for example, whether data were deduced from a single test or better value of two tests, or whether a 30 or 50 m long walkway was used. However, conclusions cannot be made regarding whether race, ethnicity, or geographical location play roles in determining 6MWT performances. Average measured 6MWT distance in our subjects (~543 m) remarkably fell within a reasonable and expected range compared with other findings.

Apart from the 6MWT performance, VO_2_ at R = 1 was measured in 46 available subjects during graded cycling exercises. Cycling was preferred instead of treadmill running primarily because of safety considerations. Treadmill running causes more stress to subjects than cycling. Therefore, cycling was preferred for old individuals and chronic disease patients. Most old Chinese subjects also had never experienced running on a treadmill, resulting in their hesitation in using the equipment. As the present study aims to provide reference data for future research in patients, using cycling in tests for such subjects provides a consistent method for future tests on chronic in-patients.

The VO_2_ test method was designed based on usual cardiovascular exercise test protocol [18,28] and on our trial tests (unpublished data). The purpose of this method was to record gradual increases in VO_2_ under time control to prevent fatigue in subjects. In our subjects, measured VO_2_ (at R = 1) values were similar to VATs of participants under the same age group according to the work of Reis et al. [32], but some yielded higher values than the others [33,34,35]. In our study, VO_2_ was tested in only 46 subjects, who showed higher physical fitness levels compared with the 60 excluded subjects. Smaller VO_2_ values would have been obtained if all 106 subjects were tested. In cardiopulmonary exercise testing, R is used in most cases to determine whether subjects reach VAT level. We observed that when R was used as the sole criterion, at R = 1 level, the VO_2_ measured was similar to the VAT. To increase efficiency, several researchers preferred measuring maximal exercise endurance (VO_2max_ or VO_2peak_) and VAT in single exercise tests, such as those conducted by Posner et al. [35] and Grigaliuniene et al. [34]. Based on our experience, this method may cause underestimation of VAT because the protocol designed for maximal exercise capacity may progress rapidly for VAT measurement. Based on the relationship between %HR_max_ and %VO_2max_ [36], HR at R = 1 (about 121 beats/min corresponding to about 75% HR_max_) in our test equals to about 60% VO_2max_; this intensity value is similar to VAT level [37]. Based on this information, we assume that our measured VO_2_ at R = 1 level falls within similar ranges with traditionally measured VAT. However, the HR after 6MWT (88 beats/min) was much lower than that at R = 1 during cycling, suggesting that exercise intensity of 6MWT distance is much lower than that of cycling exercise at R = 1. In the present study, subjects frequently engaged in walking but not cycling. Therefore, the walking test was easier for them than cycling, and this condition partly contributed to lower HR after 6MWT.

In this study, observation of 46 subjects showed highly significant correlation between 6MWT distance and VO_2_ at R = 1. This correlation may have been more evident if more subjects were involved. Strong correlation between 6MWT distance and VO_2_ at R = 1 demonstrates that 6MWT distance is a highly reliable parameter predicting exercise endurance or cardiopulmonary fitness level in similar subjects.

Apart from the factors mentioned above, muscle mass and strength evidently influence 6MWT performance. Muscle wasting, as manifested by reduction in muscle mass and loss in strength, is a common phenomenon during aging; this condition can cause decreased motor function and exercise capacity. Measurement of muscle mass poses several limitations [22]. As mentioned above, handgrip strength test is an easy and simple way to measure skeletal muscle strength. In epidemiological studies, handgrip strength is assessed more extensively than leg strength. Handgrip strength is also essential in predicting risks of heart disease and stroke [38], all-cause mortality in maintenance dialysis patients [26], and multi-morbidity among older women [39]. In our study, measured levels of handgrip strength presented similarity to those observed in older American subjects [25,40]. The significant correlation between 6MWT distance and handgrip strength also coincides with findings of previous studies on elderly adults [40]. These findings suggest that individuals who possess high handgrip strength tend to perform highly in 6MWTs. 6MWT and VO_2_ are broadly accepted as being representative of aerobic fitness, whereas measurement of handgrip strength focuses on anaerobic fitness. In this study, correlation of 6MWT with both VO_2_ and handgrip strength indicates strong association of walking performance with both aerobic capacity and muscle fitness.

Several limitations must be considered while interpreting data. First, only a portion of the subjects performed the VO_2_ test. Second, participants in our study featured various medical histories. For more specific observations, a larger number of subjects can be divided into different subgroups.

Future studies should observe these available parameters in different populations of various diseases or under various conditions (such as living in different altitudes) to gain insights into alterations in physical motor function and to explore mechanisms of improvement through effective interventions.

## 5. Conclusions

In conclusion, our study shows that 6MWT distance observed in 106 mid-aged and older Chinese home-dwelling individuals reached 543 m (559 m in males and 533 m in females) and strongly correlated with handgrip strength. During graded cycling tests on 46 subjects, VO_2_ at R = 1 correlated to 6MWT performance. Aside from providing reference values for comparison of data from diseased patients, our results suggest that 6MWT is associated with both aerobic capacity and muscle fitness.

## Figures and Tables

**Table 1:** General information on 106 subjects (AVE ± SD and ranges).

Male/Female (*n*)	Age (years)	Height (cm)	Weight (kg)	BMI (kg/m^2^)
44/62	62 ± 10 (45–90)	163 ± 7 (148–180)	66 ± 10 (45–90)	25 ± 3.2 (19–35)

AVE: average; SD: standard deviation; BMI: body mass index.

**Table 2:** HR, SBP, DBP, and RPE at pre and post six-minute walk test (6MWT).

Time Point	HR (Times/min)	SBP (mmHg)	DBP (mmHg)	RPE
pre	74.0 ± 8.9	130.3 ± 16.7	81.7 ± 9.5	9.4 ± 2.3
post	88.2 ± 11.2 **	139.5 ± 20.7 **	84.0 ± 9.7 **	11.1 ± 1.9 **

** *p* < 0.01 vs. pre (before 6MWT); HR: heart rate; SBP: systolic blood pressure; DBP: diastolic blood pressure; RPE: rate of perceived exertion.

**Table 3:** Predicting model for 6MWT distance.

Items	Coefficient (SE)	*p*	95% Confidence Interval
*r*^2^ = 0.396			
Age	−2.538 (0.53)	<0.001	−3.580 to −1.495
Handgrip strength	3.162 (0.61)	<0.001	1.955 to 4.370
Body weight	−1.978 (0.59)	0.001	−3.153 to −0.803
Constant	737.404 (50.3)	<0.001	637.640 to 837.167

## Supplementary Materials

The following are available online at http://www.mdpi.com/1660-4601/14/5/473/s1, Exercise habit and history of diseases or health problems of subjects.
