# Supplementary material for: 6MWT Performance and its Correlations with VO2 and Handgrip Strength in Home-Dwelling Mid-Aged and Older Chinese
Source: Int J Environ Res Public Health. 2017 Apr 29;14(5):473. doi: 10.3390/ijerph14050473 (PMC5451924; doi:10.3390/ijerph14050473)
Supplement: Supplementary file 1 [file ijerph-14-00473-s001.pdf]

6MWT Performance and its Correlations with VO_2 and Handgrip Strength in Home-Dwelling Mid-Aged and Older Chinese

Qing Zhang, Hailin Lu, Shiqin Pan, Yuan Lin, Kun Zhou and Li Wang

Supplementary Materials

1. Exercise Habits and History of Diseases or Health Problems of Subjects

Among 106 tested subjects, 54% (n = 57) of participants engaged in regular daily exercise while the rest did not. Walking was the most common exercise recorded. Other exercises included aerobic gymnastic, jogging, cycling, mountain climbing, Tai Chi, finger exercise (consisting of various movements, such as closing and opening of hands, squeezing of each finger, massaging specific points, and pinching of finger tips), and combined exercise modes.

A total of 59 participants presented history of diseases or operations (all operations were at least more than five years prior the present study) but were currently in stable conditions (Table S1). The questionnaire and rest BP and HR measurement were applied to ensure that the subjects may participate safely in mid-intensity physical activities without exercise contraindications or potential risk.

Table S1. History of diseases or health problems of subjects (one subject may have more than one disease or problem).

History of diseases or health problems	number (n)
Hypertension	27
Gastritis	4
Diabetes	5
Hyperlipemia	3
Cataracts	2
Lumbar muscle strain	2
Cervical spondylosis	3
Hyperthyroidism	2
Dizziness	2
Coronary heart disease	1
Incomplete right bundle branch block	1
Cholecystitis	1
Prostatic hyperplasia	1
Chronic bronchitis	1
Fatty liver	1
Gout	1
Hyperuricacidemia	1
Cholecystectomy	2
Appendectomy	1

2. Correlations of 6MWT Distance with Age, Height, Body Weight, and BMI

Age was negatively correlated with 6MWT distance (r = -0.369, p < 0.001) and work (r = -0.252, p = 0.009) (Figure. S1). Correlations of age with 6MWT distance and work were significant in male (r = -0.528, p < 0.001 and r = -0.442, p = 0.003, respectively) and in female subjects (r = -0.398, p = 0.001 and r = -0.312, p = 0.013, respectively).

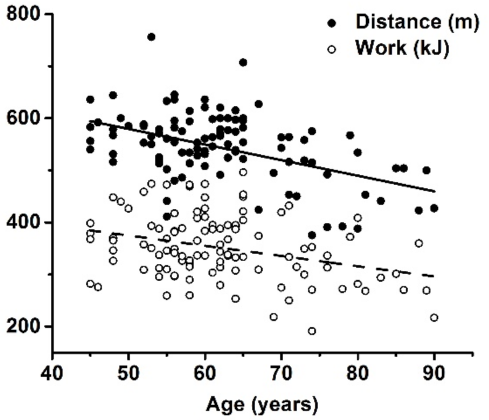

Figure S1. Correlation between age and distance (solid circles) and between age and work (hollow circles).

6MWT distance was positively correlated with body height (r = 0.193, p = 0.047), but no significant correlation was noted when males and females were separated. No correlation was observed between 6MWT distance and body weight or BMI.

3. Correlations of Handgrip Strength with Height and Body Weight

Handgrip strength was positively correlated with height (r = 0.654, $p < 0.001$) and body weight (r = 0.386, $p < 0.001$). Significance was not observed when males and females were separated.

4. Correlations of 6MWT Distance with VO_2 or Handgrip Strength in Separate Males and Females

In males, a significant positive correlation was observed between 6MWT distance and VO_2 (r = 0.694, $p < 0.001$) or between distance and relative VO_2 (r = 0.700, $p < 0.001$). In females, this correlation was almost significant (r = 0.337, p = 0.1) between 6MWT distance and VO_2 and significant between distance and relative VO_2 (r = 0.420, p = 0.036). However, after deleting one result deviating from those of 46 subjects, correlations became significant (r = 0.444, p = 0.03 for 6MWT distance and VO_2) or more significant (r = 0.500, p = 0.013 for distance and relative VO_2).

Correlation of 6MWT distance with handgrip strength was significant in males (r = 0.538, $p <$ 0.001) but not in females (r = 0.181, p = 0.175). When we deleted data of two subjects with significantly deviating results among 106 subjects, significant correlation was also noted between 6MWT distance with handgrip strength in females (r = 0.278, p = 0.038).

5. General Information and Fitness Parameters in 46 Participants Who Participated in VO_2 Test

Data of 46 participants who participated in VO_2 test possibly differed from those remaining 60 participants. To properly interpret results, general and fitness parameters of 46 participants, as listed in Table S2, were compared with data of 60 participants who did not participate in VO_2 test. Participants (46 in total) who performed the VO_2 test were younger than the remaining participants and exhibited larger handgrip strength and 6MWT distance. No difference in body weight, height, and BMI were observed (Table S2).

Table S2. General and fitness parameters of 46 subjects who participated in the VO_2 test compared with 60 subjects who were excluded (AVE ± SD).

Subjects (n)†	Age (years)	Height (cm)	Weight (kg)	BMI (kg/m^2)	Handgrip strength (kg)	6MWT distance (m)
46[a]	57.9 ± 8.2**	163.0 ± 6.4	67.1 ± 9.0	25.3 ± 3.0	32.1 ± 10.2*	575.9 ± 43.6**
60[b]	65.3 ± 10.8	162.3 ± 7.1	65.2 ± 10.1	24.8 ± 3.4	28.2 ± 8.9	518.4 ± 71.6

†: [a] "46" is the number of subjects who participated in VO_2 test, and [b] "60" is the number of participants who refused to participate; ** $p < 0.001$ and * $p < 0.05$ *vs.* corresponding data from 60 subjects who did not participate in VO_2 tests.
